# Development of Thiazolidinedione-Based HDAC6 Inhibitors to Overcome Methamphetamine Addiction

**DOI:** 10.3390/ijms20246213

**Published:** 2019-12-09

**Authors:** Chiranjeev Sharma, Yong Jin Oh, Byoungduck Park, Sooyeun Lee, Chul-Ho Jeong, Sangkil Lee, Ji Hae Seo, Young Ho Seo

**Affiliations:** 1 College of Pharmacy, Keimyung University, Daegu 42601, Korea; 1chiranjeevsharma1@gmail.com (C.S.); ldp7899@naver.com (Y.J.O.); bdpark@kmu.ac.kr (B.P.); sylee21@kmu.ac.kr (S.L.); chjeong75@kmu.ac.kr (C.-H.J.); skdavid@kmu.ac.kr (S.L.); 2 Department of Biochemistry, School of Medicine, Keimyung University, Daegu 42601, Korea; seojh@kmu.ac.kr

**Keywords:** HDAC6 inhibitors, thiazolidinedione, methamphetamine, drug addiction

## Abstract

Thiazolidinedione is a five-membered heterocycle that is widely used in drug discovery endeavors. In this study, we report the design, synthesis, and biological evaluation of a series of thiazolidinedione-based HDAC6 inhibitors. In particular, compound **6b** exerts an excellent inhibitory activity against HDAC6 with an IC_50_ value of 21 nM, displaying a good HDAC6 selectivity over HDAC1. Compound **6b** dose-dependently induces the acetylation level of *α*-tubulin via inhibition of HDAC6 in human neuroblastoma SH-SY5Y cell line. Moreover, compound **6b** efficiently reverses methamphetamine-induced morphology changes of SH-SY5Y cells via regulating acetylation landscape of *α*-tubulin. Collectively, compound **6b** represents a novel HDAC6-isoform selective inhibitor and demonstrates promising therapeutic potential for the treatment of methamphetamine addiction.

## 1. Introduction

Methamphetamine (METH) is a widely abused illicit psychostimulant that causes euphoria, hyperactivity, and wakefulness. METH abuse has dramatically risen over the last decade owing to its readily accessibility and powerful euphoric effect [1]. Acute overdose of METH induces cardiac arrhythmia, strokes, seizures, and hyperthermia, and repeated exposure to this substance leads to severe addiction, altered cognitive function, and neurological damage [2,3,4,5,6,7]. This chronic METH abuse is accompanied by significant changes in gene and protein expression within specific brain subregions such as dorsal striatum in the reward circuitry, suggesting that there is a clear connection between epigenetic mechanisms and METH addiction [8]. Therefore, a number of studies have been conducted to elucidate the epigenetic mechanisms underlying METH addiction [9,10]. Interestingly, it is reported that acute and chronic exposure to METH increases histone acetylation, implying that epigenetic enzymes including histone deacetylases (HDACs) participate in the mechanisms of METH addiction [11,12,13]. Moreover, it is known that administration of METH regulates cytoplasmic HDAC6 enzyme, altering epigenetic landscape of *α*-tubulin in endothelial cells [14]. Given the fact that HDAC enzymes play an important role in METH addition, we decided to investigate the therapeutic potential of HDAC inhibitors for the treatment of METH addiction. 

HDAC is an enzyme that catalyzes the removal of acetyl groups from lysine residues at the N-terminal tails of histones [15]. These enzymes also regulate the acetylation status of non-histone proteins such as *α*-tubulin, Hsp90, and p53 [16]. There are eighteen isoforms of HDAC enzymes, further classified into four classes. These four identified classes are characterized by their distinct functions, substrate specificities, and subcellular locations. Accordingly, there has been great effort to develop isoform-selective HDAC inhibitors to elucidate the detailed functions of each isoform and to avoid unwanted side effects in chemotherapy [17,18].

## 2. Results and Discussion

**Figure 1:**
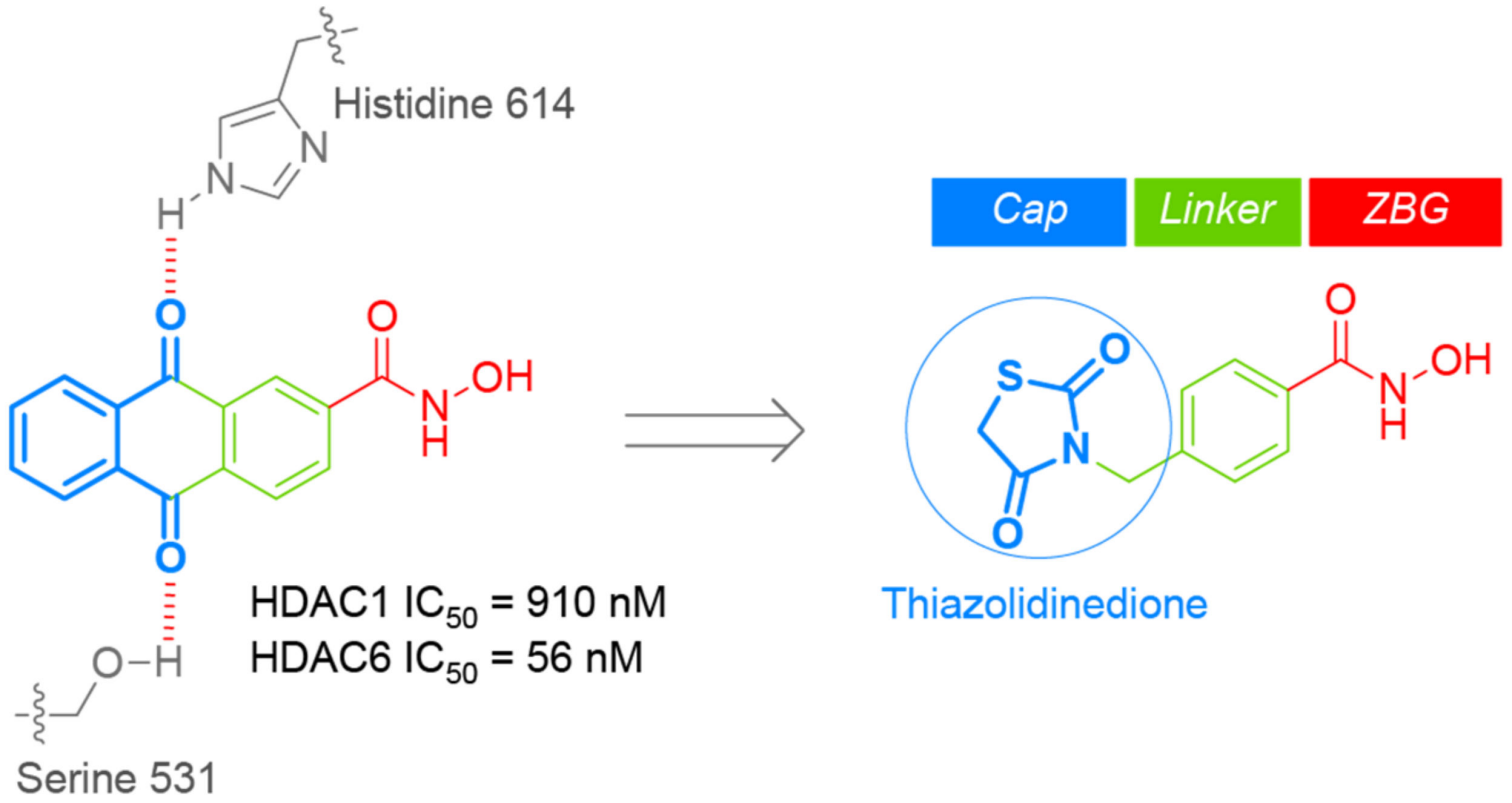
Drug design of thiazolidinedione-based histone deacetylase 6 (HDAC6) inhibitors.

We previously performed a structure-based virtual screening and discovered a novel class of anthraquinone-based HDAC6 inhibitors with a good HDAC6 selectivity over other HDAC isozymes (Figure 1) [19]. We found out that two carbonyl oxygens of anthraquinone played a pivotal role in exhibiting selective inhibition of HDAC6. These carbonyl groups recognized the unique rim and surface area of HDAC6, which is different from other HDAC isozymes. Despite the fact that this anthraquinone-based HDAC6 inhibitor displayed an excellent in vitro biological activity, its intrinsic poor solubility hampered further study for in vivo application. Thiazolidinedione (TZD) is a five-membered ring molecule containing two heteroatoms (nitrogen and sulfur) and two carbonyl groups. Thiazolidinedione and its derivatives have diverse biological activities including anti-microbial, anti-oxidant, anti-inflammatory, anti-diabetic, anti-cancer, and anti-tubercular activities, and a number of thiazolidinedione-based drugs, such as troglitazone and rosiglitazone, have been clinically approved for the treatment of type 2 diabetes [20,21]. The fact that thiazolidinedione is a privileged scaffold from clinically approved drugs with two carbonyl groups prompted us to explore the thiazolidinedione-based HDAC6 inhibitors. 

**Scheme 1:**
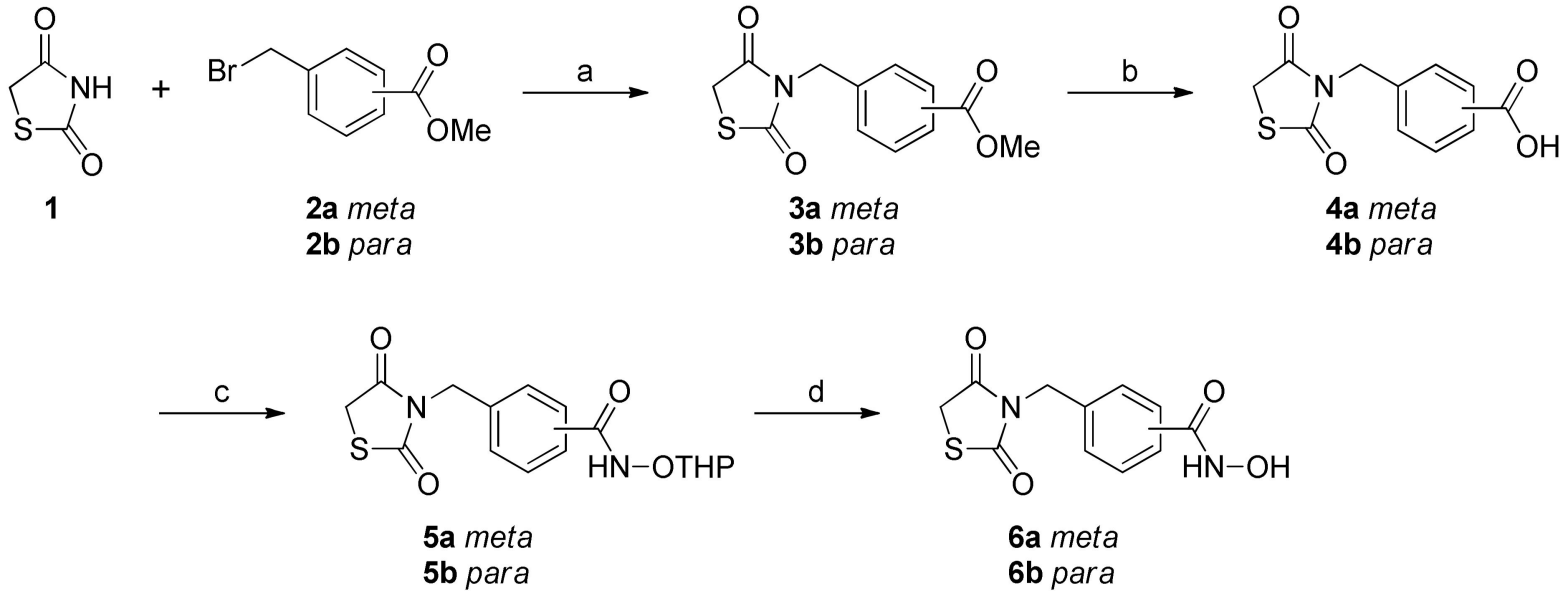
Synthesis of compounds **6a-b^a^**. ^a^ Reagents and conditions: (**a**) K_2_CO_3_, acetone, reflux, 12 h, 53% for **3a**, 55% for **3b**; (**b**) 6N HCl reflux, 12 h, 76% for **4a**, 89% for **4b**; (**c**) EDC·HCl, HOBt, NH_2_OTHP, TEA, rt, 18 h, 43% for **5a**, 57% for **5b**; (**d**) HCl, Et_2_O, DCM, rt, 2 h, 36% for **6a**, 42% for **6b**.

The synthesis of thiazolidinedione-based HDAC6 inhibitors **6a-b** is illustrated in Scheme 1. The key intermediate **3a-b** were synthesized following the previously reported procedure [22]. Briefly, the base-promoted N-alkylation reaction of thiazolidine-2,4-one (**1**) with methyl 3-(bromomethyl)benzoate (**2a**) or methyl 4-(bromomethyl)benzoate (**2b**) successfully provided compounds **3a-b** in 53–55% yields. The initial attempt to directly convert ester **3a-b** to hydroxamate **6a-b** using hydroxylamine in the presence of sodium hydroxide in methanol was not fruitful due to the instability of thiazolidine-2,4-one ring in this reaction condition. That led us to follow an alternative synthetic route. Therefore, compound **3a-b** were hydrolyzed into carboxylic acid **4a-b** in the aqueous acidic condition. Having prepared carboxylic acid **4a-b**, we then performed amide coupling reaction of **4a-b** with NH_2_OTHP using EDC, HOBt, and TEA in DCM, which provided compound **5a-b** in 43–57% yields. Finally, the removal of THP-protecting group from compound **5a-b** was accomplished by the acidic hydrolysis and successfully afforded compound **6a-b** in 36–42% yields.

With compound **6a-b** in hand, we first measured in vitro inhibitory activity of meta-analogue **6a** and para-analogue **6b** against HDAC1 and HDAC6 enzymes. As shown in Table 1, para-analogue **6b** led to higher binding affinity to HDAC1 and HDAC6 than meta-analogue **6a**, in that para-analogue **6b** furnished IC_50_ values of 388 nM and 21 nM against HDAC1 and HDAC6, respectively. This result directed us to synthesize more para-analogues derived from compound **6b**.

**Scheme 2:**
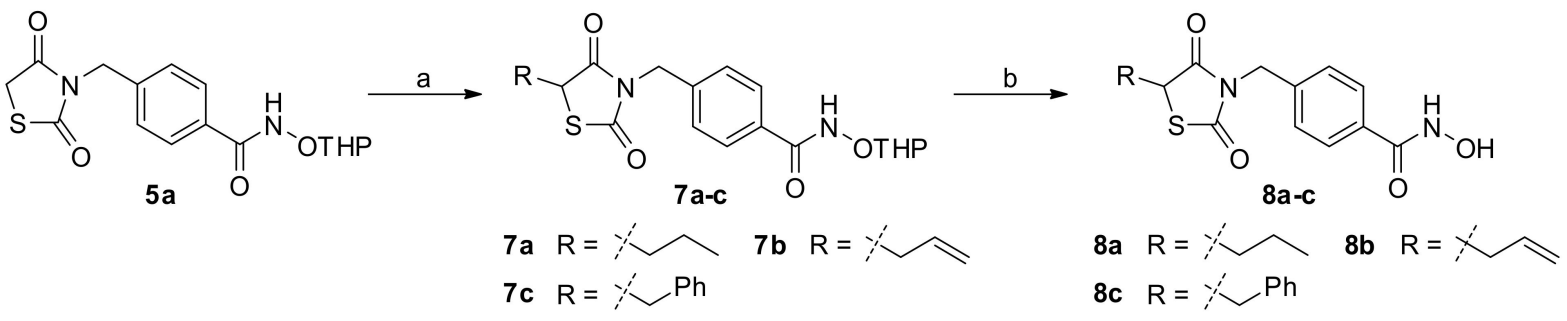
Synthesis of compounds **8a-c^a^**. ^a^ Reagents and conditions: (**a**) R-Br, K_2_CO_3_, DMF, rt, 18 h, 14% for **7a**, 27% for **7b**, 23% for **7c** (**d**) HCl in Et_2_O, DCM, rt, 2h, 38% for **8a**, 40% for **8b**, 44% for **8c**.

The synthetic route to compounds **8a-c** as shown in Scheme 2 started from the intermediate **5a**. Base-catalyzed alkylation reaction of **5a** with allyl bromide, propyl iodide, and benzyl bromide in DMF led to compounds **7a-c** in 14–27% yields. Subsequently, the cleavage of THP-protecting group from compounds **7a-c** under acidic condition resulted in compounds **8a-c** in 38–44% yields.

Upon completion of synthesis, we examined inhibitory activities of 5-substituted thiazolidinedione-based HDAC inhibitors **8a-c** against HDAC1 and HDAC6 isotypes. As shown in Table 1, 5-substituted thiazolidinedione-based HDAC inhibitors **8a-c** led to a decrease in their binding affinity to both HDAC1 and HDAC6 isotypes, compared with their non-substituted parent compound **6b**. The result indicated that the substitution of 2,4-thiazolidinedione at 5-position caused a negative effect on their proper binding to HDAC1 and HDAC6 enzymes. Interestingly, compound **6b** (HDAC6 IC_50_ = 21 nM) displayed an excellent inhibitory activity against HDAC6, even better than FDA-approved drug SAHA (HDAC6 IC_50_ = 226 nM). Overall, compound **6b** exhibited the most excellent inhibitory activity against HDAC1 and HDAC6 among newly synthesized analogues with a good HDAC6 selectivity over HDAC1. Accordingly, we chose compound **6b** for further biological evaluation. 

**Figure 2:**
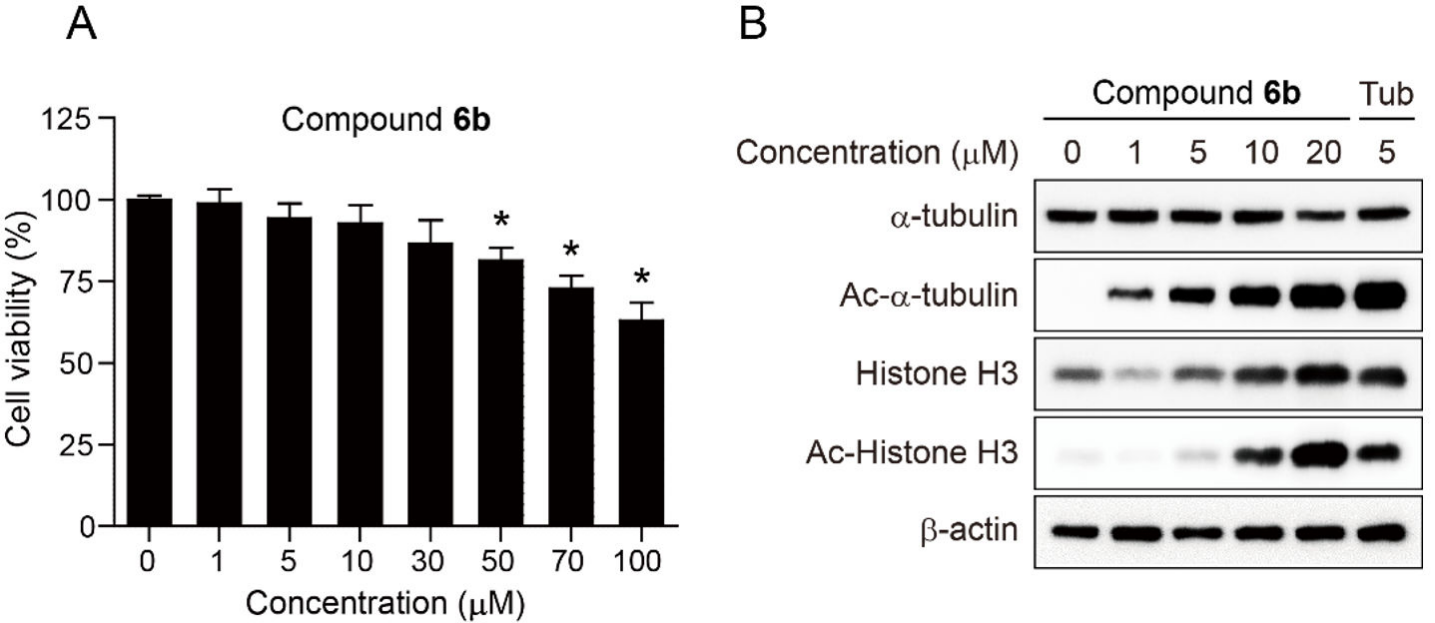
Effect of compound **6b** on the cell viability of SH-SY5Y and the acetylation status of *α*-tubulin and Histone H3. (**A**) SH-SY5Y cells were treated with the indicated concentrations of compound **6b** for 24 h and the cell viability was measured using the colorimetric MTS assay. Data are presented as mean ± SD (*n* = 4). (**B**) SH-SY5Y cells were incubated with the indicated concentrations of compound **6b** or Tubastatin A (Tub) for 24 h and the expression levels of *α*-tubulin, Ac-*α*-tubulin, Histone H3, and Ac-Histone H3 were analysed by Western blot. Tubastatin A was employed as a positive control and *β*-actin was used as a loading control. The *p* value was obtained by Student *t*-test. * *p* < 0.01 as compared to vehicle.

We next evaluated the effect of compound **6b** on the cell viability of SH-SY5Y, which is a human neuroblastoma cell line (Figure 2). Although the exposure of SH-SY5Y cells with high concentrations (50, 70, and 100 µM) of **6b** decreased the cell viability of SH-SY5Y in a dose-dependent manner, compound **6b** exhibited no cytotoxic effect on cell viability up to 30 µM concentration. It has been well-reported that selective inhibition of HDAC6 isoform did not produce a lethal effect on cell viability, in contrast to inhibition of class I HDAC enzymes. 

We further explored the precise cellular mechanism of compound **6b**. Histone H3 and *α*-tubulin are well-known substrates of HDAC1 and HDAC6 [23,24]. Therefore, inhibition of HDAC1 and HDAC6 enzymes promotes the accumulation of acetylated Histone H3 and *α*-tubulin, respectively. Therefore, SH-SY5Y cells were incubated with compound **6b** at various concentrations (0, 1, 5, 10, and 20 µM) for 24 h and the expression levels of *α*-tubulin, Ac-*α*-tubulin, Histone H3, and Ac-Histone H3 were analysed by Western blot. Highly selective HDAC6 inhibitor, Tubastatin A (Tub, 5 µM) was employed as a reference drug. As expected, compound **6b** dose-dependently induced the acetylation of *α*-tubulin and Histone H3. The administration of **6b** at relatively low concentration of 1 µM caused the acetylation of *α*-tubulin via inhibition of HDAC6. In contrast, 10 µM concentration of **6b** was able to promote the acetylation of Histone H3 via inhibition of HDAC1, suggesting that compound **6b** more efficiently inhibited HDAC6 than HDAC1 in this cellular setting. It is also worth noting that the protein level of Histone H3 increases in proportion to the concentration of compound **6b**, implying that compound **6b** might have an impact on the transcription factors. The expression level of internal standard, *β*-actin remained unchanged as expected. Taken together, the results indicated that compound **6b** more potently suppressed HDAC6 enzyme activity than HDAC1 in SH-SY5Y cells, which is positively correlated with in vitro HDAC assay shown in Table 1. 

**Figure 3:**
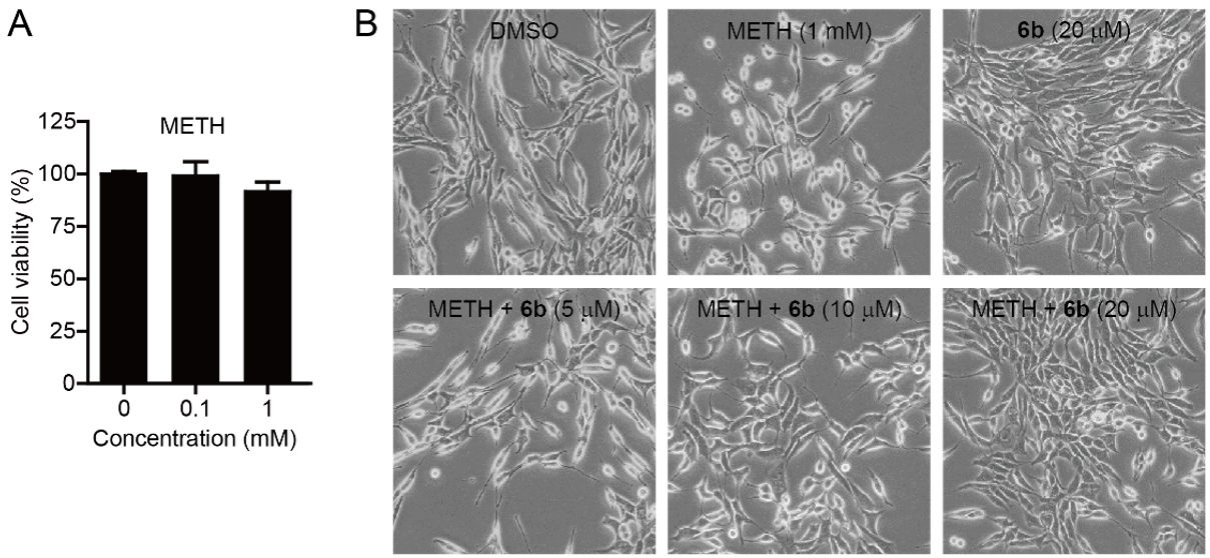
Effect of methamphetamine (METH) and compound **6b** on the cell morphology and viability of SH-SY5Y cells. (**A**) Effect of methamphetamine on the cell viability of SH-SY5Y. Cells were incubated with the indicated concentrations of methamphetamine (METH) for 24 h and cell viability was measured using the colorimetric MTS assay. Data are presented as mean ± SD (*n* = 4). (**B**) Bright-field images of SH-SY5Y cells. Cells were pretreated with methamphetamine (METH, 1 mM) for 4 h and then incubated with the indicated concentrations of compound **6b** for 24 h.

There has been a report that methamphetamine promotes the acetylation of *α*-tubulin via the modification of HDAC6 activity, impacting cytoskeleton stability, cell motility, and polarity [14]. This cytoskeleton disarrangement is strongly associated with the disruption of blood brain barrier (BBB) integrity, allowing harmful substances in the bloodstream to leak into the central nervous system (CNS) [25]. This study prompted us to investigate the effect of methamphetamine on cell morphology. We first examined whether methamphetamine exerted any cytotoxic effect on cell viability prior to analysing the effect of methamphetamine (METH) on the cell morphology of SH-SY5Y cells (Figure 3). SH-SY5Y cells were incubated with methamphetamine (0, 0.1, and 1 mM) for 24 h and then analysed the cell viability using MTS assay. The assay revealed that methamphetamine exerted no toxicity up to 1 mM concentration for 24 h. We next investigated the effect of METH on the cell morphology and the reverse effect of compound **6b** in preventing METH-induced cell morphology changes. Accordingly, SH-SY5Y cells were pretreated with 1 mM of METH for 4 h and then incubated with compound **6b** (5, 10, and 20 µM) for 24 h. As expected, untreated SH-SY5Y cells (DMSO) had a normal elongated spindle morphology. Upon the treatment of cells with 1 mM of METH, a majority of SH-SY5Y cells showed a rounded shape with a shrunken morphology. Subsequently, the administration of compound **6b** (5, 10, and 20 µM) led to a reversed effect on the cell morphology change from a rounded shape back to a spindle shape in a dose-dependent manner. 

**Figure 4:**
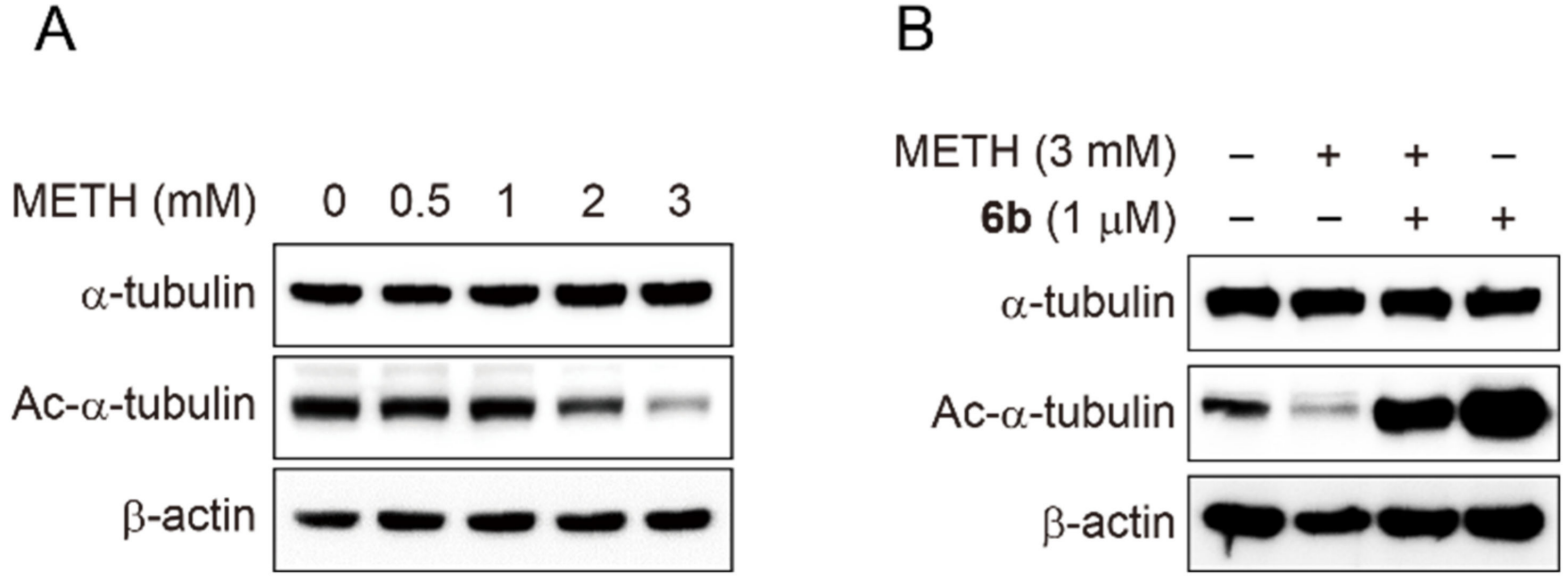
Western blot analysis of *α*-tubulin acetylation status after the treatment with methamphetamine (METH) and compound **6b**. (**A**) SH-SY5Y cells were incubated with the indicated concentrations of methamphetamine (METH) for 24 h and the expression levels of Ac-*α*-tubulin and *α*-tubulin were analysed by Western blot. *β*-actin was used as a loading control. (**B**) SH-SY5Y cells were pretreated with methamphetamine (METH, 3 mM) for 4 h before exposure to compound **6b** (1 µM) for 24 h. The expression levels of Ac-*α*-tubulin and *α*-tubulin were analysed by Western blot. *β*-actin was used as a loading control.

We next explored the underlying molecular and biochemical mechanisms behind the morphological changes. *α*-Tubulin is the basic protein that constitutes the cytoskeleton and the dynamic acetylation and deacetylation of *α*-tubulin play an important role in the morphology of cells. SH-SY5Y cells were treated with methamphetamine at various concentrations (0, 0.5, 1, 2, and 3 mM) for 24 h and the expression levels of *α*-tubulin and Ac-*α*-tubulin were measured by Western blot (Figure 4). The result indicated that methamphetamine dose-dependently decreased the acetylation of *α*-tubulin, while the expression level of *α*-tubulin remained unchanged. We next investigated if compound **6b** reversed the loss of the acetylation caused by methamphetamine. Therefore, SH-SY5Y cells were treated with methamphetamine (METH, 3 mM) for 4 h, followed by incubation with compound **6b** for 24 h. As seen in Figure 4, treatment of cells with METH reduced the acetylation status of *α*-tubulin. Interestingly, the subsequent treatment of cells with compound **6b** led to a significant increase in the acetylation of *α*-tubulin by reversing the METH-promoted loss of acetylation. Collectively, the results indicated that METH promoted the morphological changes of cells by disrupting the acetylation status of *α*-tubulin, which could be reversed by compound **6b** probably via the inhibition of HDAC6 enzymes. 

**Figure 5:**
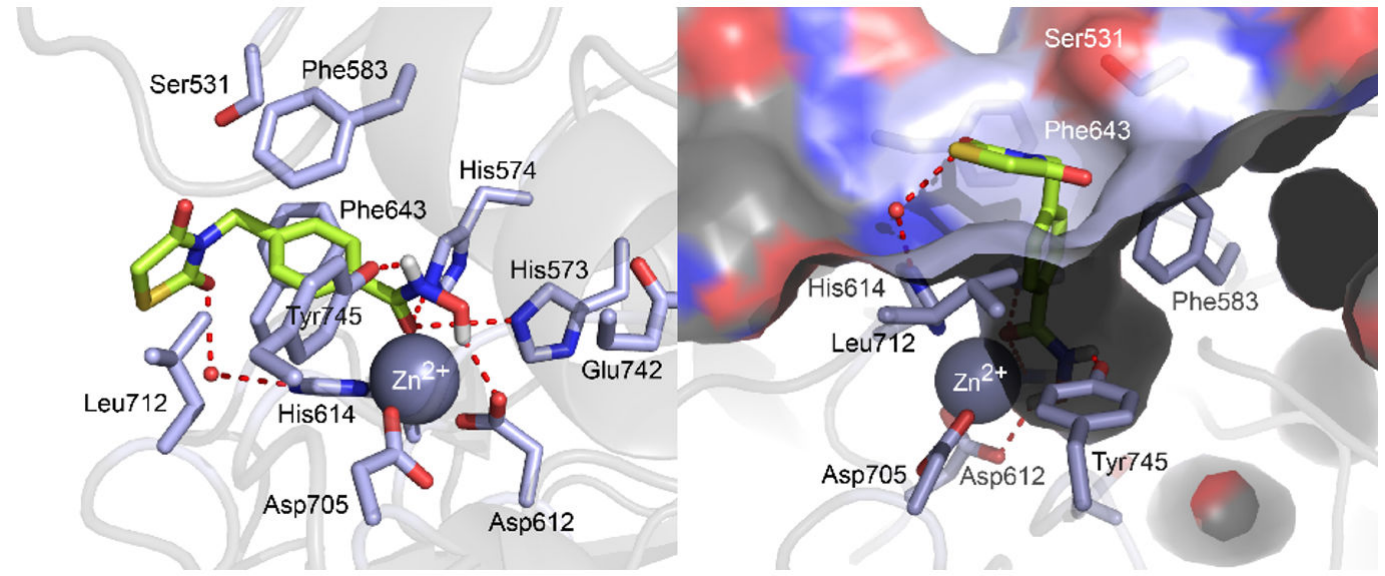
Molecular docking pose of compound **6b** in the binding pocket of HDAC6 (PDB code: 5EF7). The carbon, oxygen, nitrogen, sulfur, and hydrogen atoms of compound **6b** are shown in lime, red, blue, yellow, and white, respectively. The side chains of the binding pocket are colored by atom type (carbon, light blue; oxygen, red; nitrogen, red) and labeled with their residue name. Water molecule is shown as a red sphere. The hydrogen bonds are shown in dashed lines. Molecular docking simulations were performed by Autodock 4.2 and docking poses were visualized using PyMOL1.3.

To investigate the molecular basis of interactions between compound **6b** and HDAC6 (PDB code: 5EF7), we performed in silico docking study (Figure 5 and Supplementary Figure S1). The docking simulation suggested that compound **6b** well bound to the substrate binding pocket, validating its inhibitory potential to HDAC6. The hydroxamate group of compound **6b** chelated the Zn^2+^ ion in the bottom of the pocket with its carbonyl oxygen (C=O) and hydroxyl oxygen (OH) in a bidentate fashion.

Additionally, the carbonyl oxygen (C=O) of the hydroxamate formed two hydrogen bonds with the side chain of His573 and His574, while the hydroxyl (OH) group and amine (NH) group of the hydroxamate formed hydrogen bonds with Asp612 and Tyr745, respectively. The middle phenyl ring of compound **6b** were nicely positioned in the hydrophobic channel, forming π−π interactions with the lipophilic side chains of Phe583 and Phe643 residues. Thiazolidinedione ring was located in the rim of the substrate binding pocket, in that one carbonyl oxygen (C=O) of thiazolidinedione ring formed a hydrogen bonding interaction with a conserved water molecule and the hydrophobic sulfur and carbon atoms at the 1 and 5 position of the thiazolidinedione ring formed proximal Van der Waals interactions with the lipophilic side chain of Leu712. Collectively, the docking study illustrated that compound **6b** well fitted into the active site of HDAC6 with diverse intermolecular interactions such as zinc chelation, hydrogen bonds, π−π interactions, and Van der Waals interactions and the estimated binding energy of compound **6b** resulted in −7.02 kcal/mol. 

## 3. Materials and Methods 

### 3.1. Chemistry

#### 3.1.1. General Methods and Materials

All reagents and solvents were purchased from commercial suppliers and used without further purification. All experiments dealing with moisture-sensitive compounds were carried out under argon atmosphere. Concentration or solvent removal under reduced pressure was carried out using rotary evaporator. Analytical thin layer chroatography was performed on precoated silica gel F254 TLC plates (E, Merck) with visualization under UV light or by staining using iodine. Column chromatography and medium pressure liquid chromatography (MPLC) was conducted on silica (Merck Silica Gel 40–63 µm) or performed by using a Biotage SP1 flash purification system with prepacked silica gel cartridges (Biotage). NMR analyses were carried out using a JNM-ECZ500R (500 MHz) manufactured by Jeol resonance. Chemical shifts are reported in parts per million (δ). The deuterium lock signal of the sample solvent was used as a reference, and coupling constants (J) are given in hertz (Hz). The splitting pattern abbreviations are as follows: s, singlet; d, doublet; t, triplet; q, quartet; dd, doublet of doublet; td, triplet of doublet; m, multiplet. The LC-QTOF-MS analysis was performed using an Agilent 6530 Accurate-Mass Q-TOF LC/MS System with Agilent 1290 Infinity LC (Agilent Technologies, Palo Alto, CA, USA). The guard column and the analytical column were Zorbax SB-C8 (3.5 μm, 2.1 × 30 mm, Agilent Technologies) and Zorbax SB-Aq (1.8 μm, 2.1 × 100 mm, Agilent Technologies), respectively, and were maintained at 40 °C. The mobile phase consisted of 0.1% formic acid in water (A) and 0.1% formic acid in acetonitrile (B). The gradient conditions were as follows: 0–30 min, 1–20% B; 30–40 min, 20–90% B; 40–45 min, 90% B; 45–47 min, 90–1% B; 47–52 min, 1% B at a flow rate of 400 μL/min. The MS system was operated using ESI in the positive and the negative ionization mode. The optimized conditions of the QTOF-MS system for both ionization modes were as follows: drying gas temperature, 300 °C; drying gas flow, 10 L/min; nebulization pressure, 45 psi; sheath gas temperature, 350 °C; sheath gas flow, 10 L/min; capillary voltage, 3500 V; nozzle voltage, 0 V; fragmentor voltage, 175 V; skimmer voltage, 65 V. The mass range was 50–1700 m/z and the scan rate was 2.00 spectra/sec for both MS and MS/MS analyses.

#### 3.1.2. General Procedure for the Synthesis of Compounds **3a-b**

The mixture of thiazolidine-2,4-one (10 mmol), methyl 4-(bromomethyl)benzoate or methyl 3-(bromomethyl)benzoate (11 mmol), and anhydrous K_2_CO_3_ (15 mmol) was refluxed in acetone (50 mL) overnight. The solid K_2_CO_3_ was filtered and the filtrate was evaporated to obtain the crude product, which was purified by MPLC to afford compound **3a-b** in 53–55%.

##### Methyl 3-((2,4-dioxothiazolidin-3-yl)methyl)benzoate (**3a**) 

53% Yield. ^1^H-NMR (500 MHz, CDCl_3_) δ 8.04 (s, 1H), 7.99 (d, *J* = 7.4 Hz, 1H), 7.59 (d, *J* = 7.4 Hz, 1H), 7.41 (t, *J* = 7.7 Hz, 1H), 4.81 (s, 2H), 3.97 (s, 2H), 3.91 (s, 3H).

##### Methyl 4-((2,4-dioxothiazolidin-3-yl)methyl)benzoate (**3b**) 

55% Yield. ^1^H-NMR (500 MHz, CDCl_3_) δ 7.99 (d, *J* = 8.6 Hz, 2H), 7.44 (d, *J* = 8.0 Hz, 2H), 4.81 (s, 2H), 3.97 (s, 2H), 3.91 (s, 3H).

#### 3.1.3. General Procedure for the Synthesis of Compounds **4a-b**

A suspension of compound **3a** or **3b** (2 mmol) in 6N HCl (25 mL) was stirred at reflux for 12 h. The mixture was then cooled and kept at 4 °C for 2 h. The desired product precipitated which was filtered, washed with water (2 × 20 mL) and dried in vacuo to afford compound **4a-b** in 76–89%. 

##### 3-((2,4-Dioxothiazolidin-3-yl)methyl)benzoic acid (**4a**)

76% Yield. ^1^H-NMR (500 MHz, CD_3_OD) δ 7.99 (s, 1H), 7.95 (d, *J* = 7.4 Hz, 1H), 7.58 (d, *J* = 7.4 Hz, 1H), 7.44 (t, *J* = 7.4 Hz, 1H), 4.81 (s, 2H), 4.13 (s, 2H).

##### 4-((2,4-Dioxothiazolidin-3-yl)methyl)benzoic acid (**4b**) 

89% Yield. ^1^H-NMR (500 MHz, CD_3_OD) δ 7.98 (d, *J* = 8.0 Hz, 2H), 7.42 (d, *J* = 8.0 Hz, 2H), 4.81 (s, 2H), 4.15 (s, 2H)

#### 3.1.4. General Procedure for the Synthesis of Compounds **5a-b**

To a solution of compound **4a** or **4b** (1.1 mmol), EDC·HCl (4.4 mmol), HOBt (2.2 mmol) in dry DCM was added triethylamine (7.7 mmol) and O-tetrahydropyran-2-ylhydroxylamine (1.4 mmol). The reaction mixture was stirred at room temperature for 18 h. Then, DCM was washed with brine solution. The organic layer was dried over Na_2_SO_4_, concentrated in vacuo. The product was purified by MPLC to afford **5a-b** in 43–57% yield.

##### 3-((2,4-Dioxothiazolidin-3-yl)methyl)-N-((tetrahydro-2H-pyran-2-yl)oxy)benzamide (**5a**) 

43% Yield. ^1^H-NMR (500 MHz, CDCl_3_) δ 9.20 (s, 1H), 7.71 (s, 1H), 7.69 (d, *J* = 8.0 Hz, 1H), 7.50 (d, *J* = 7.4 Hz, 1H), 7.37 (t, *J* = 7.7 Hz, 1H), 5.06 (s, 1H), 4.76 (s, 2H), 3.98 (d, *J* = 11.5 Hz, 1H), 3.96 (s, 2H), 3.62 (t, *J* = 5.7 Hz, 1H), 1.81–1.87 (m, 3H), 1.56–1.65 (m, 3H).

##### 4-((2,4-Dioxothiazolidin-3-yl)methyl)-N-((tetrahydro-2H-pyran-2-yl)oxy)benzamide (**5b**) 

57% Yield. ^1^H-NMR (500 MHz, CDCl_3_) δ 8.86 (s, 1H), 7.71 (d, *J* = 8.0 Hz, 2H), 7.44 (d, *J* = 8.0 Hz, 2H), 5.06 (s, 1H), 4.79 (s, 2H), 3.99 (d, *J* = 8.6 Hz, 1H), 3.96 (s, 2H), 3.64 (dd, *J* = 6.3, 5.2 Hz, 1H), 1.83–1.92 (m, 3H), 1.59–1.66 (m, 3H).

#### 3.1.5. General Procedure for the Synthesis of Compounds **7a-c**

Compound **6a** (0.22 mmol), alkyl halide (0.22 mmol) and anhydrous K_2_CO_3_ (0.22 mmol) were added into dry DMF (5 mL) and the mixture was stirred at room temperature for 18 h. Then DMF was evaporated in vacuo. The solid crude product was purified by MPLC to afford **7a-c** in 14–27% yield.

##### 4-((2,4-Dioxo-5-propylthiazolidin-3-yl)methyl)-N-((tetrahydro-2H-pyran-2-yl)oxy)benzamide (**7a**)

14% yield. ^1^H-NMR (500 MHz, CDCl_3_) δ 8.74 (s, 1H), 7.71 (d, *J* = 8.0 Hz, 2H), 7.43 (d, *J* = 8.0 Hz, 2H), 5.06 (s, 1H), 4.77 (dd, *J* = 20.6, 14.3 Hz, 2H), 4.21 (q, *J* = 4.4 Hz, 1H), 3.97–4.01 (m, 1H), 3.65 (t, *J* = 5.4 Hz, 1H), 2.15 (td, *J* = 9.5, 5.3 Hz, 1H), 1.79–1.91 (m, 4H), 1.59–1.68 (m, 3H), 1.38–1.50 (m, 2H), 0.95 (t, *J* = 7.2 Hz, 3H).

##### 4-((5-Allyl-2,4-dioxothiazolidin-3-yl)methyl)-N-((tetrahydro-2H-pyran-2-yl)oxy)benzamide (**7b**) 

27% yield. ^1^H-NMR (500 MHz, CDCl_3_) δ 9.09 (s, 1H), 7.70 (d, *J* = 8.0 Hz, 2H), 7.35–7.39 (m, 2H), 5.66–5.75 (m, 1H), 5.13–5.18 (m, 2H), 5.05 (s, 1H), 4.75 (dd, *J* = 24.1, 14.3 Hz, 2H), 4.26–4.30 (m, 1H), 3.99 (q, *J* = 10.1 Hz, 1H), 3.62 (t, *J* = 5.4 Hz, 1H), 2.87–2.92 (m, 1H), 2.56–2.63 (m, 1H), 1.81–1.87 (m, 3H), 1.53–1.64 (m, 3H).

##### 4-((5-Benzyl-2,4-dioxothiazolidin-3-yl)methyl)-N-((tetrahydro-2H-pyran-2-yl)oxy)benzamide (**7c**) 

23% yield. ^1^H-NMR (500 MHz, CDCl_3_) δ 8.81 (s, 1H), 7.69 (d, *J* = 8.0 Hz, 2H), 7.31 (d, *J* = 8.0 Hz, 2H), 7.27 (s, 3H), 7.17 (q, *J* = 2.9 Hz, 2H), 5.08 (s, 1H), 4.74 (dd, *J* = 22.6, 14.6 Hz, 2H), 4.50 (q, *J* = 4.4 Hz, 1H), 3.98–4.02 (m, 1H), 3.67 (t, *J* = 5.7 Hz, 1H), 3.49 (dd, *J* = 14.3, 4.0 Hz, 1H), 3.14 (dd, *J* = 14.3, 9.2 Hz, 1H), 1.85–1.91 (m, 3H), 1.62–1.68 (m, 3H).

#### 3.1.6. General Procedure for the Synthesis of Compounds **6a-b** and **8a-c**

Compounds **5a-b** or **7a-c** (0.1 mmol) were dissolved in CH_2_Cl_2_ (4 mL). Then 2M HCl in diethyl ether (4 mL) was added dropwise. The reaction mixture was stirred for 2h at room temperature. The solvent was evaporated in vacuo. The crude was purified by MPLC to afford **6a-b** and **8a-c** in 36–44% yield.

##### 3-((2,4-Dioxothiazolidin-3-yl)methyl)-N-hydroxybenzamide (**6a**) 

36% yield. ^1^H-NMR (500 MHz, CD_3_OD) δ 7.71 (s, 1H), 7.66 (d, *J* = 8.0 Hz, 1H), 7.52 (d, *J* = 8.0 Hz, 1H), 7.43 (t, *J* = 7.7 Hz, 1H), 4.80 (s, 2H), 4.13 (s, 2H) ^13^C-NMR (125 MHz, CD_3_OD) δ 173.96, 173.50, 167.80, 137.75, 132.73, 130.01, 127.98, 127.64, 45.55, 34.75. ESI MS (m/z) 267.04 [M+H]^+^.

##### 4-((2,4-Dioxothiazolidin-3-yl)methyl)-N-hydroxybenzamide (**6b**) 

42% yield. ^1^H-NMR (500 MHz, CD_3_OD) δ 7.71 (d, *J* = 8.0 Hz, 2H), 7.42 (d, *J* = 8.0 Hz, 2H), 4.79 (s, 2H), 4.13 (s, 2H). ^13^C-NMR (125 MHz, CD_3_OD) δ 173.88, 173.43, 167.72, 140.76, 133.13, 129.44, 128.44, 45.48, 34.72. ESI MS (m/z) 267.04 [M+H]^+^.

##### 4-((2,4-Dioxo-5-propylthiazolidin-3-yl)methyl)-N-hydroxybenzamide (**8a**) 

38% yield. ^1^H-NMR (500 MHz, CD_3_OD) δ 7.98 (d, *J* = 8.0 Hz, 2H), 7.41 (d, *J* = 8.6 Hz, 2H), 4.80 (s, 2H), 4.48–4.51 (m, 1H), 2.08–2.15 (m, 1H), 1.83–1.90 (m, 1H), 1.34–1.53 (m, 2H), 0.94–0.97 (m, 3H). ^13^C-NMR (125 MHz, CD_3_OD) δ 176.20, 172.88, 169.34, 142.09, 131.57, 131.09, 129.17, 50.89, 45.51, 35.87, 20.97, 13.76 ESI MS (m/z) 309.09 [M+H]^+^.

##### 4-((5-Allyl-2,4-dioxothiazolidin-3-yl)methyl)-N-hydroxybenzamide (**8b**) 

40% yield. ^1^H-NMR (500 MHz, CD_3_OD) δ 7.98 (d, *J* = 8.6 Hz, 2H), 7.41 (t, *J* = 7.7 Hz, 2H), 5.73–5.81 (m, 1H), 5.11–5.19 (m, 2H), 4.77–4.83 (m, 2H), 4.59 (q, *J* = 4.0 Hz, 1H), 2.87–2.91 (m, 1H), 2.62–2.68 (m, 1H) ^13^C-NMR (125 MHz, CD_3_OD) δ 175.56, 172.79, 169.34, 142.04, 133.49, 131.55, 131.05, 129.22, 120.10, 50.33, 45.53, 37.38. ESI MS (m/z) 307.08 [M+H]^+^.

##### 4-((5-Benzyl-2,4-dioxothiazolidin-3-yl)methyl)-N-hydroxybenzamide (**8c**) 

^1^H-NMR (500 MHz, CD_3_OD) 44% yield. δ 7.92 (d, *J* = 8.0 Hz, 2H), 7.18–7.24 (m, 7H), 4.83 (q, *J* = 4.0 Hz, 1H), 4.73 (dd, *J* = 22.3, 14.9 Hz, 2H), 3.41 (dd, *J* = 14.0, 4.3 Hz, 1H), 3.26 (q, *J* = 7.3 Hz, 1H). ^13^C-NMR (125 MHz, CD_3_OD) δ 175.37, 172.60, 169.39, 141.84, 136.97, 131.38, 131.03, 130.84, 129.54, 128.96, 128.44, 52.28, 45.47, 38.47. ESI MS (m/z) 357.09 [M+H]^+^.

### 3.2. Biology

#### 3.2.1. Materials

Dulbecco’s Modified Eagle’s medium (DMEM) with L-glutamine was purchased from GenDEPOT (Barker, TX, USA) and fatal bovine serum (FBS) and penicillin and streptomycin were purchased Gibco BRL (Gaithersburg, MD, USA). Antibodies for *α*-tubulin, Ac-*α*-tubulin (Lys40), Histone H3, Ac-Histone H3 (Lys9), and *β*-actin were purchased from Cell Signaling Technology (Boston, MA, USA). Goat anti-rabbit IgG horseradish peroxidase conjugate was purchased from Santa Cruz Biotechnology (Santa Cruz, CA, USA). Cell Titer 96 Aqueous One Solution cell proliferation assay kit was purchased from Promega (Madison, WI, USA). Amersham ECL select Western blotting detection reagent was purchased from GE Healthcare. HDAC fluorogenic assay kits (HDAC1, #50061; HDAC6, #50076) were purchased from BPS Bioscience (San Diego, CA, USA). Tubastain A (Portland, OR, USA) was purchased from TCI chemicals. For in vitro studies, Methamphetamine (METH) was purchased from the Ministry of Food and Drug Safety (Cheongju, Korea).

#### 3.2.2. Cell Culture 

SH-SY5Y were grown in DMEM supplemented with streptomycin (500 mg/mL), penicillin (100 units/m), and 10% fetal bovine serum (FBS). The cells were incubated at 37 °C in a humidified atmosphere containing 5% CO_2_.

#### 3.2.3. Assessment of Cell Morphology 

SH-SY5Y cells (1 × 10^4^ cells/well) were seeded in 6-well plate, and the cells were allowed to attach for 72 h. Culture medium was then changed to fresh medium containing METH (1 mM) for pretreat 4 h. After being preincubated for 4 h, add to compound **6b** (5, 10, and 20 µM) for 24 h. Cell morphology was observed with inverted phase contrast microscope (Olympus, Tokyo, Japan) at 20× objective

#### 3.2.4. HDAC Assay 

Enzymatic HDAC assay was performed following manufacturer’s protocol (BPS Bioscience). Briefly, HDAC assay buffer (35 µL) was mixed with 5 µL of BSA (1 mg/mL) and 5 µL of HDAC substrate (200 µM) in 96-well black plate. Five microliters of HDAC1 enzyme (0.4 ng/μL) and HDAC6 enzyme (7 ng/µL) were added to the each well, followed by various concentrations of compound **6a-b**, **8a-c** and SAHA (5 µL), and then resulting mixture was incubated at 37 °C for 30 min. After incubation, 50 µL of undiluted 2 × HDAC developer was added to each well. After the mixture was incubated at RT for 15 min, fluorescence intensity was measured using a microplate reader at 360 nm excitation and 460 nm emission wavelengths.

#### 3.2.5. Cell Proliferation Assay

SH-SY5Y (1 × 10^3^ cells/well) were seeded in 96-well plate, the medium volume was brought to 100 µL, and the cells were allowed to attach for 14 h. Various concentrations of compound **6b** (1, 5, 10, 30, 50, 70, and 100 µM) and METH (0.1 and 1 mM) were then added to the wells. Cells were then incubated at 37 °C for 24 h. Cell viability was determined using the Promega Cell Titer 96 Aqueous One Solution cell proliferation assay. Absorbance at 490 nm was read on Tecan Infinite F200 Pro plate reader, and values were expressed as percent of absorbance from cells incubated in DMSO alone. 

#### 3.2.6. Western Blot

The cells were washed and lysed in ice-cold lysis buffer (23 mM Tris-HCl pH 7.6, 130 mM NaCl, 1% NP-40, 1% sodium deoxycholate, 0.1% SDS) and 30 µg of lysate per lane was separated by SDS-PAGE, followed by transferring to a PVDF membrane (Bio rad, Hercules, USA). The membrane was blocked with 5% skim milk in TBST, and then incubated with primary antibody. After being treated with goat-anti rabbit secondary antibody (Santa Cruz, CA, USA) coupled horseradish peroxidase, proteins were visualized by ECL chemiluminescence according to the instructions of the manufacturer’s instruction (GE Healthcare, Chicago, IL, USA).

#### 3.2.7. Statistical Analysis 

Experiments were performed at least three times, with consistent results. The results are given as mean ± standard derivation (SD). The *p*-value was assessed using Student *t*-tests. Results were considered statistically significant at *p* < 0.01.

### 3.3. Docking Studies

In silico docking of compound **6b** with the 3D coordinates of the X-ray crystal structure of HDAC6 (PDB code: 5EF7) was accomplished using the AutoDock 4.2 program downloaded from the Molecular Graphics Laboratory of the Scripps Research Institute. The AutoDock program was chosen because it uses a genetic algorithm to generate the poses of the ligand inside a known or predicted binding site utilizing the Lamarckian version of the genetic algorithm where the changes in conformations adopted by molecules after in situ optimization are used as subsequent poses for the offspring. In the docking experiments carried out, Gasteiger charges were placed on the X-ray structure of HDAC6 along with 6b using tools from the AutoDock suite. A grid box centered on the substrate binding pocket of HDACs enzyme with definitions of 50 × 50 × 50 points and 0.375 Å spacing was chosen for ligand docking experiments. The docking parameters consisted of setting the population size to 150, the number of generations to 27,000, and the number of evaluations to 2,500,000, while the number of docking runs was set to 100 with a cutoff of 1 Å for the root-mean-square tolerance for the grouping of each docking run. The docking pose of HDAC6 with compound **6b** was depicted in Figure 5 and rendering of the picture was generated using PyMol (DeLanoScientific). Furthermore, we performed the self-docking experiment of the native ligand HPOB into HDAC6 and assessed the effectiveness of the docking simulations. The estimated binding energy of HPOB resulted in −7.85 kcal/mol and RMSD between the docked binding pose and the native binding pose led to 1.8 Å.

## 4. Conclusions

In the current study, we designed and synthesized a new series of thiazolidinedione-based HDAC6 inhibitors. Biological evaluation of these analogues illustrated that compound **6b** furnished the most potent HDAC6 inhibition activity with an IC_50_ value of 21 nM, which is approximately 10-fold greater than FDA-approved drug, SAHA (HDAC6 IC_50_ = 226 nM). Exposure of SH-SY5Y cells with compound **6b** more efficiently promoted the acetylation of *α*-tubulin than Histone H3, indicating that compound **6b** suppressed the activity of HDAC6 enzyme more selectively than HDAC1. Furthermore, compound **6b** reversed methamphetamine-induced morphology changes of SH-SY5Y cells in a dose-dependent manner. Western blot analysis revealed that biochemical mechanisms underlying methamphetamine-induced morphology changes are associated with disturbance of *α*-tubulin acetylation. Taken together, compound **6b** represents a novel HDAC6-selective inhibitor, demonstrating promising therapeutic potential in methamphetamine addiction. 

## Figures and Tables

**Table 1:** In vitro inhibitory activity against HDAC1 and HDAC6 isotypes.

Compound	Structure	HDAC1 (IC_50_; nM)	HDAC6 (IC_50_; nM)	Selectivity Index ^a^
**6a**	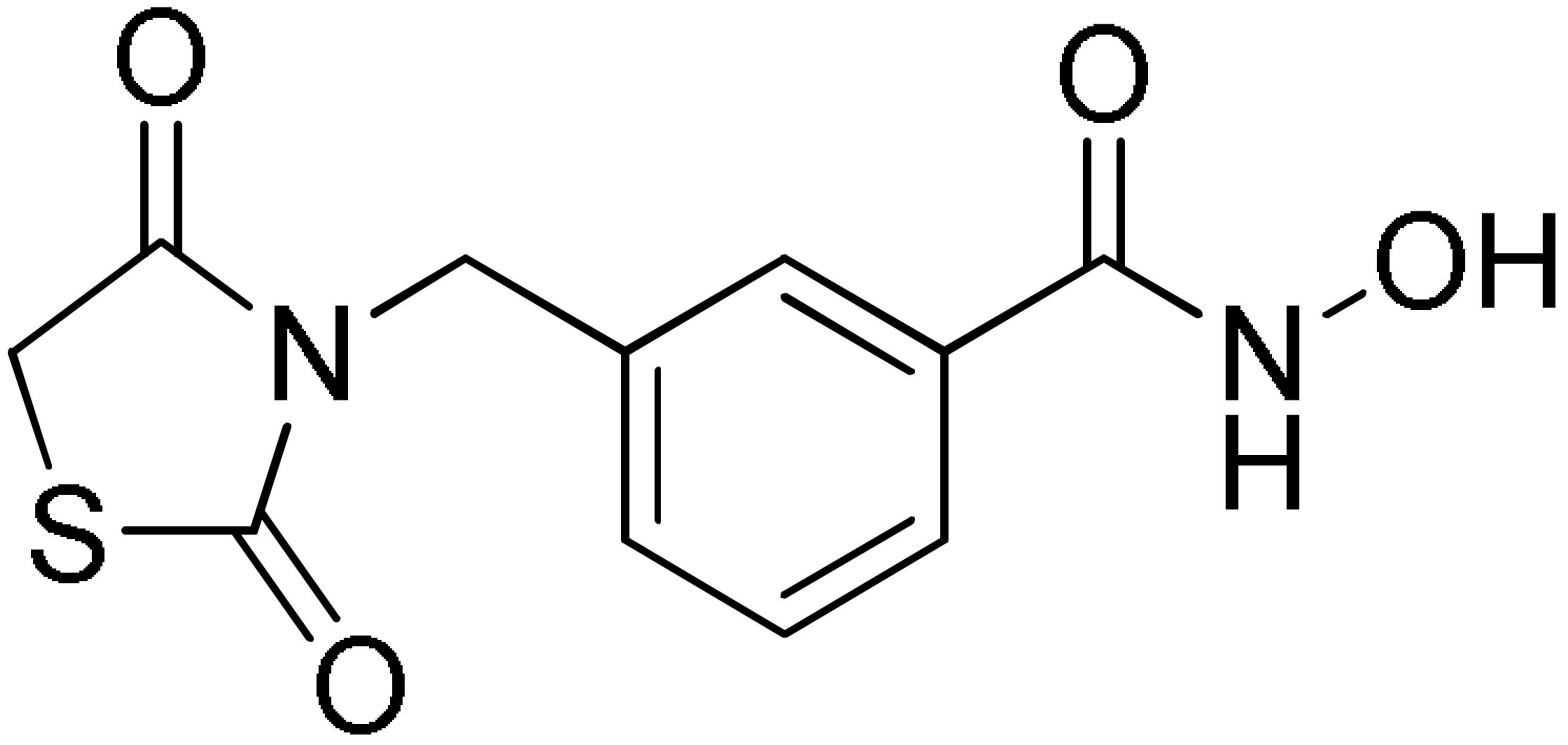	>50,000	1961	NA
**6b**	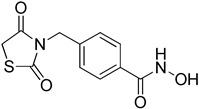	388	21	18.5
**8a**	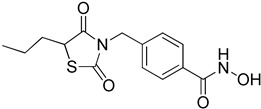	>100,000	79,290	NA
**8b**	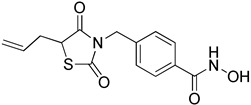	>100,000	1302	NA
**8c**	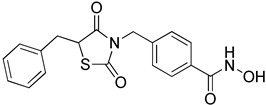	>100,000	628	NA
**SAHA**	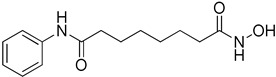	236	226	1.0

^a^ Selectivity index: the ratio of HDAC1 over HDAC6 isotype. NA: not available.

## Supplementary Materials

Supplementary materials can be found at https://www.mdpi.com/1422-0067/20/24/6213/s1.
