# Supplementary material for: Development of Thiazolidinedione-Based HDAC6 Inhibitors to Overcome Methamphetamine Addiction
Source: Int J Mol Sci. 2019 Dec 9;20(24):6213. doi: 10.3390/ijms20246213 (PMC6940941; doi:10.3390/ijms20246213)
Supplement: Supplementary file 1 [file ijms-20-06213-s001.pdf]

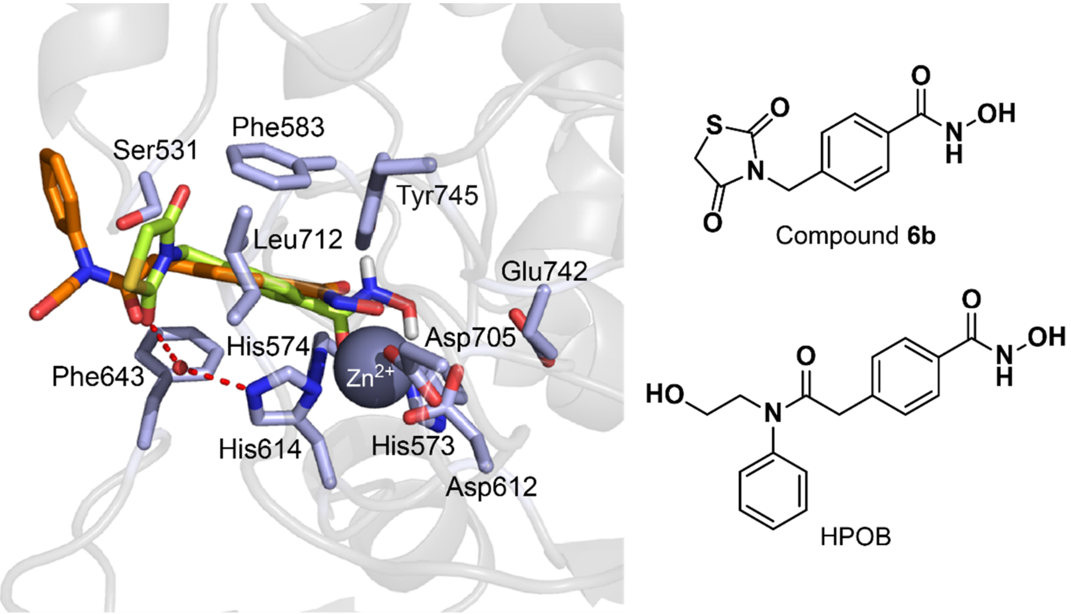

Supplementary Figure S1. Superimposition of crystallographic structure of HPOB and docked pose of compound **6b** in HDAC6 (PDB code: 5EF7). The carbon atoms of HPOB and compound **6b** are depicted in orange and lime, respectively. The oxygen, nitrogen, sulfur, and hydrogen atoms of HPOB and compound **6b** are shown in red, blue, yellow, and white, respectively. The side chains of the binding pocket are colored by atom type (carbon, light blue; oxygen, red; nitrogen, red) and labeled with their residue name. Water molecule is shown as a red sphere. The hydrogen bonds are shown in dashed lines. Molecular docking simulations were performed by AutoDock 4.2 and docking poses were visualized using PyMOL1.3.
